# Assessment of Tumor-Associated Tissue Eosinophilia (TATE) and Tumor-Associated Macrophages (TAMs) in Canine Transitional Cell Carcinoma of the Urinary Bladder

**DOI:** 10.3390/ani14030519

**Published:** 2024-02-05

**Authors:** Rita Files, Victor Okwu, Nuno Topa, Marisa Sousa, Filipe Silva, Paula Rodrigues, Leonor Delgado, Justina Prada, Isabel Pires

**Affiliations:** 1 Department of Veterinary Sciences, University of Trás-os-Montes and Alto Douro, 5000-801 Vila Real, Portugal; ritafiles2000@gmail.com (R.F.); apharmvic@gmail.com (V.O.); tsa.nuno.topa@gmail.com (N.T.); marisa.fc.sousa@gmail.com (M.S.); fsilva@utad.pt (F.S.); pavelar@utad.pt (P.R.); jprada@utad.pt (J.P.); 2 Animal and Veterinary Research Centre (CECAV), Associate Laboratory for Animal and Veterinary Sciences (AL4AnimalS), University of Trás-os-Montes and Alto Douro, 5000-801 Vila Real, Portugal; 3 UNIPRO—Oral Pathology and Rehabilitation Research Unit, University Institute of Health Sciences—CESPU (IUCS-CESPU), 4585-116 Gandra, Portugal; leonordelgado@inno.pt; 4 Pathology Department, INNO Specialized Veterinary Services, 4710-503 Braga, Portugal

**Keywords:** transitional cell carcinoma, eosinophilia, macrophages, urinary bladder, canine, immune checkpoints

## Abstract

**Simple Summary:**

Transitional cell carcinoma of the urinary bladder in dogs is a severe type of cancer with difficult treatment and recovery. These dog cancers are quite similar to bladder cancers in humans, making them a good comparison for study. This research looked into how certain types of immune cells, specifically eosinophils (referred to as tumor-associated tissue eosinophils) and macrophages (tumor-associated macrophages), affect the development and severity of these cancers. This study examined 34 cases of dog bladder cancer and used specific staining techniques to identify eosinophils and macrophages. The tumors were divided into two groups based on their aggressiveness (low- and high-grade). It was found that the less severe cancers had more eosinophils, while the more severe ones had more macrophages. This suggests that the presence of these immune cells is linked to how aggressive the cancer is. This study proposes that targeting these cells could be a new way to treat this type of cancer in dogs, which might also provide insights into human cancer treatment.

**Abstract:**

Transitional cell carcinoma of the urinary bladder is a significant neoplasm in dogs, characterized by a poor prognosis and a high metastatic potential. These canine spontaneous tumors share many characteristics with human transitional cell carcinoma, making them an excellent comparative model. The role of inflammatory infiltration in tumor development and progression is frequently contradictory, especially concerning tumor-associated tissue eosinophils (TATE) and tumor-associated macrophages (TAMs). This study aims to analyze TATE and TAMs in canine transitional cell carcinoma of the urinary bladder. Congo Red staining was used to identify TATE, and immunohistochemistry was performed to detect TAMs in 34 cases of canine transitional cell carcinoma of the bladder carcinomas, categorized into low and high grades. Statistically significant differences were observed between the number of eosinophils and macrophages in the two groups of tumors. The number of TATE was higher in low-grade malignant tumors, but the number of TAMs was higher in high-grade tumors. Our findings suggest the importance of TATEs and TAMs in the aggressiveness of canine transitional cell carcinoma and propose their potential use as therapeutic targets.

## 1. Introduction

Transitional cell carcinoma (TCC), also known as urothelial carcinoma, emerges as the most prevalent form of urinary bladder cancer in dogs, impacting tens of thousands of animals globally each year [1,2]. Potentially invasive, canine TCC poses significant challenges, including urinary tract obstruction, distant metastases occurring in over 50% of cases, and clinical signs that raise concerns for dogs and their owners. Various risk factors, such as exposure to older flea control products, lawn chemicals, obesity, female gender, and association with specific breeds, contribute to TCC incidence [3,4,5].

The classification of canine TCC includes low-grade superficial tumors and high-grade invasive tumors. Notably, canine invasive transitional tumors are more prevalent than humans, affecting over 50,000 dogs annually in the USA. Diagnosing TCC in dogs presents challenges due to nonspecific clinical signs, leading to delayed detection, with over 10% of cases presenting metastatic disease at diagnosis, contributing to an unfavorable clinical prognosis [6,7,8,9].

In humans, approximately 20% of bladder cancers are diagnosed as high-grade invasive TCC [2,4]. In dogs, more than 90% of cases are intermediate to high-grade tumors. TCC in dogs could be regarded as a relevant model for human studies, especially in more aggressive tumors, sharing similarities in environmental risk factors, clinical presentation, pathophysiological characteristics, genetic and epigenetic regulation, metastatic behavior, and response to therapies [2,4,10].

TCC typically occurs in older dogs, with mean and median ages reported at the time of diagnosis ranging from 9 to 11 years, although the disease can manifest earlier in a minority of dogs. TCC most frequently localizes to the trigone region of the bladder, and papillary lesions and thickening of the bladder wall may cause partial or complete urinary tract obstruction. Moreover, TCC has been reported to involve the urethra in 56% of dogs and the prostate in 29% of male dogs [4,11]. Breed-related risk also plays a significant role, with certain breeds exhibiting a greater predisposition, including Fox Terriers, Scottish Terriers, Shetland Shepherds, West Highland White Terriers, Keeshonds, Samoyeds, Beagles, Collies, Airedale Terriers, American Eskimo dogs, Chesapeake Bay Retrievers, and Schipperkes [2,4,10].

The tumor microenvironment (TME) consists of immune cells, fibroblasts, endothelial cells, and cancer cells, playing a crucial role in cancer progression and metastasis formation. Each immune cell, whether innate or adaptive, infiltrates the TME to modulate tumor growth, resulting in different outcomes [12,13].

Circulating monocytes and macrophages are recruited to tumors throughout tumor progression, crucial in modifying the tumor microenvironment to expedite tumor progression. Macrophages adjust their functional phenotypes in response to various microenvironmental signals from tumor and stromal cells. Based on their morphological, phenotypic, and functional heterogeneity, macrophages are categorized into two main subtypes: M1 and M2. M1 macrophages play an essential role in the anti-tumor immune response, primarily mediating pro-inflammatory processes in the tumor microenvironment [14,15]. Conversely, M2 macrophages exhibit protumor characteristics, promoting tumor growth and metastatic dissemination [16]. When coexisting with a small population of M1 macrophages, M2 macrophages form “tumour-associated macrophages” (TAMs), playing a crucial role in the TME and influencing tumor growth. The infiltration of TAMs into the TME is associated with unfavorable clinical outcomes in several types of cancer, reducing the effectiveness of conventional treatments [17,18,19].

The modulation of TAMs has become a promising area of research for innovative therapies. Various strategies can be employed for therapeutic intervention, such as blocking the CCL2–CCR2 or CCR5–CCL5 axes, depleting TAMs by blocking CSF-1 or CSF-1R, blocking macrophage checkpoint inhibitors like CD47/SIRP1α, PD-1/PD-L1, MHCI/LILRB1, and CD24/Siglec-10, and suppressing the pro-tumor activity of macrophages (by inhibiting TGF-β or VEGF). Macrophage depletion or inhibition using CCL2, CSF-1, and CSF-1R inhibitors has shown efficacy against mouse and human tumors [20,21]. Although there are still only a few studies in dogs, specific therapies targeting macrophages, such as Liposomal clodronate, are beginning to emerge [22]. Losartan, a type I angiotensin II receptor blocker, has seen renewed interest due to its macrophage-targeting properties. These drugs act as specific inhibitors of the CCL2-CCR2 axis [23]. Additionally, other methods to re-polarize tumor-associated macrophages (TAMs) have been explored in canine studies. These include using a canine monoclonal agonist antibody against CD40, a macrophage-activating receptor [24], and treatments like paclitaxel, imatinib, BTK inhibitors, and chloroquine [25]. Also, checkpoint inhibitors are promising in veterinary oncology [26,27,28].

As for eosinophils, their functions can be grouped into four main categories: effector functions, tissue remodeling, cellular interactions, and immunomodulation. These constituents of the innate immune system play essential roles in antibacterial, antiviral, antiparasitic, and anti-tumor activities. The differentiation of eosinophils is regulated by cytokines such as IL-3, GM-CSF, SCF, and, notably, IL-5. The interaction of eosinophils with tumor cells and the tumor microenvironment (TME) occurs through the release of cytokines and granular content, exerting an influence on the tumor environment. Moreover, the binding of eosinophil receptors to tumor cells and the TME alters cancer outcomes [29,30]. Emerging therapeutic strategies, including immune checkpoint inhibitors and chimeric antigen receptor (CAR) T-cell therapies, show promising potential in cancer treatment [31].

This study investigated the presence of tumor-associated tissue eosinophilia (TATE), using the red Congo staining technique, and tumor-associated macrophages (TAMs) through the immunohistochemical expression of MAC387 (a newly infiltrated tissue macrophages marker) in canine transitional cell carcinoma. The aim was to analyze the potential association of these elements with tumor aggressiveness and assess whether there is a correlation between TATE and TAMs.

## 2. Materials and Methods

### 2.1. Animals and Histopathology

This study includes 34 canine transitional cell carcinoma and 10 samples from normal dog urinary bladders used as a normal control for comparison with bladder carcinoma samples. The tumor samples were retrieved from the Histology and Pathology Laboratory archives at the University of Trás-os-Montes e Alto Douro. These tumors were obtained in surgical procedures, cystoscopy, or during necropsy, previously fixed in 10% buffered formalin and paraffin-embedded. Clinical information, including age, gender, and breed, was documented for each animal in the study.

The microscopic analysis involved the staining of 3 μm-thick sections with hematoxylin and eosin. Two distinct pathologists (Isabel Pires, Justina Prada) independently reviewed each sample. The analysis encompassed all slides, meticulously evaluating every section conducted.

Histopathologic diagnosis was based on the World Health Organization (WHO) histological classification of animal tumors [32].

A Nikon Eclipse E600 microscope with a Nikon DXM1200 digital camera (Nikon Instruments Inc., Melville, NY, USA) was used for microscopical observations and image capture.

### 2.2. Histological Grading

Grading systems have been outlined for urinary bladder transitional cell carcinoma in dogs, yet without regular use by pathologists and, consequently, without implementation in clinical practice or correlation with survival. In our study, following the initial analysis and diagnosis, canine transitional cell carcinoma was histologically categorized into low-grade and high-grade, as described in Table 1, adapted from [33]. This classification draws on the system devised by Cheng in 2012 for human bladder urothelial carcinomas, albeit simplified into two groups, mirroring the system proposed by the WHO in 2004 and 2016 for humans. However, unlike the WHO’s 2004 framework, tumor invasiveness is underscored as a distinctive criterion for high-grade TCC of urinary bladder in this veterinary grading system [33,34,35].

### 2.3. TATE Evaluation in Canine TCC

#### 2.3.1. Congo Red Staining Procedure

Congo Red staining was employed to visualize and quantify tumor-associated eosinophils, following the protocol outlined by Joshi and Kaijkar [36]. The sections were dewaxed in xylene and underwent gradual rehydration using ethanol. Distilled water was used to wash the sections, which were stained with a 0.5% Congo Red alcohol solution for 10 min. Subsequently, a 0.2% alcoholic potassium hydroxide solution was applied, followed by a wash and staining with hematoxylin. The sections were dehydrated in ascending ethanol concentrations, deparaffinized with xylol, and ultimately mounted with Entellan (Merck KGaA, Darmstadt, Germany).

We used a grade 1 mast cell tumor, recognized for its notable eosinophil infiltrate, to be a positive control.

#### 2.3.2. Quantification of TATE

To assess the presence of eosinophils, tissues were examined using 400× magnification. Eosinophil counts were conducted in the primary field and nine adjacent fields, with the total count recorded as eosinophils per 10 high-power fields (HPF). Only eosinophils that met specific criteria, such as nucleated cells with intensely red cytoplasmic mixtures, were included. Red blood cells overlapping with other cells or eosinophils confined to lymphovascular spaces were excluded from the count. The assessment of the number of eosinophils in the tumor was categorized as low, moderate, or high, following the criteria outlined in Table 2, used in previous publications [37].

### 2.4. TAMs Evaluation in Canine TCC

#### 2.4.1. Immunohistochemistry Staining Procedure

TAMs were assessed through the immunohistochemical evaluation of MAC387+ macrophages using the NovolinkTM polymer detection system (Leica Biosystems^®^, Newcastle, UK). Briefly, samples were dewaxed over 15 min, followed by a hydration sequence using a series of alcohol solutions (100%, 95%, 80%, and 70%), with each step lasting 5 min. Antigen retrieval was performed by immersing the samples in a 10 mM citrate buffer (pH 6.0 ± 0.2) and subjecting them to three 5 min cycles in a 750 W microwave. To inhibit peroxidase, a 3% hydrogen peroxide solution was applied for 30 min, followed by treatment of the sections with a protein block for 5 min. Primary antibody incubation was carried out with the MAC387 antibody (AbDSerotec, MorphoSys UK Ltd., Kidlington, Oxford, UK; Clone MCA 874G) at a 1:100 dilution in PBS overnight at 4 °C. A post-primary reagent was then applied, followed by the application of Novolink Polymer, each for 30 min. After PBS washes, the samples were developed through a 10 min incubation with 3,3-diaminobenzidine (DAB) and then counterstained with Gill’s hematoxylin for one minute. The sections were washed with water, dehydrated in a series of alcohol solutions (95%, 95%, 100%, 100%) for 3 min at each stage, cleared in xylene, and finally mounted using Entellan mounting medium (Merck^®^). The antibody has demonstrated cross-reaction in canine tissue samples [38,39].

The positive control for MAC387 consisted of canine epidermal and liver tissues, while the negative control involved omitting the primary antibody in PBS.

#### 2.4.2. Quantification of TAMs

Quantitative assessment of macrophage immunoexpression, identified through MAC387 positivity, was conducted by observing a brownish coloration in the cell cytoplasm, considering nuclear morphology to distinguish polymorphonuclear neutrophils. Two independent observers (I.P. and J.P.) evaluated immunoreactivity without knowing the tumor grade.

TAMs were quantified in the three areas with positive cells and counted across ten high-power fields (HPF) at 400× magnification within these areas. Based on the counts, TAMs were classified into three scores, as described previously in [39] (Table 3).

### 2.5. Statistical Analysis

For statistical analysis, chi-square tests (χ^2^) were conducted using IBM SPSS Statistics, version 21, to explore potential associations between variables. The results were presented as absolute and relative frequencies. Significance levels were established at *p* < 0.05.

## 3. Results

### 3.1. Clinical Information

In total, 47.1% (16 cases) of the animals were female, while 52.9% (18 cases) were male. The age of the animals varied from 4 to 15 years, with an average of 10.59 ± 2.85 years. Regarding canine breeds, our findings revealed that mixed-breed dogs had the highest number of cases (24 out of 34), followed by Poodle (4 out of 34) and Labrador Retriever (3 out of 34), with Castro Laboreiro Dog (2 out of 34) and Serra da Estrela Dog (1 out of 34) also being represented.

### 3.2. Histological Grading of the Canine TCC of the Urinary Bladder

The classification was assigned based on the criteria described above, and 7 cases were classified as low-grade tumors, and 27 cases were classified as high-grade tumors.

### 3.3. TATE Was Increased in High-Grade Canine TCC

In CR staining, eosinophils are easily identifiable by their brightly red-stained cytoplasm against a dark blue background of other tissue structures. In the normal urinary bladder, eosinophils were either absent or present in scarce numbers (score 1, less than 4 in 10 HPF).

Among the total of 34 tumors, the majority (21 out of 34) exhibited a low score of TATE, 5 cases (14.7%) had a moderate TATE value, and 8 cases (23.5%) displayed intense infiltration (score 3).

**Figure 1:**
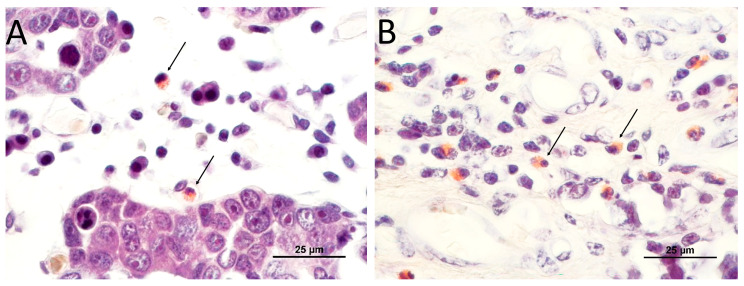
TATE colored by Congo Red Staining (arrows) in canine TCC: (**A**) low score of TATE (1) in high-grade transitional cell carcinoma; (**B**) high score (3) of TATE in low-grade canine transitional cell carcinoma.

**Figure 2:**
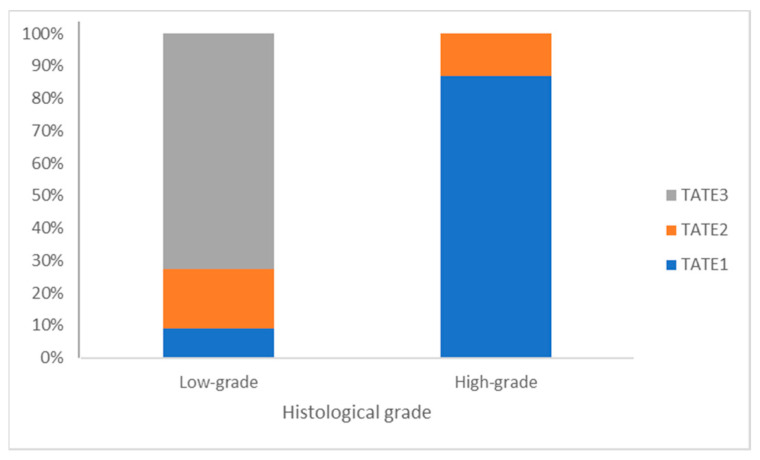
TATE in low-grade and high-grade canine transitional cell carcinomas.

When analyzed in the two categories of aggressiveness, low-grade and high-grade, statistically significant differences (*p* < 0.001) were observed in the TATE score. TATE were lower in high-grade tumors. Therefore, in low-grade malignant tumors, eosinophils were either moderate (18.2%) or abundant (72.7%). Only one low-grade transitional cell carcinoma (9.01%) showed low TATE infiltration. Conversely, in high-grade malignant tumors, eosinophils were predominantly low (87.0%), as represented in Figure 1 and Figure 2.

### 3.4. TAMs Were Increased in High-Grade Tumors Canine TCC

The immunohistochemical analysis aimed to identify and quantify MAC387-positive macrophages revealed positive staining not only in macrophages but also in neutrophils and, in certain instances, in neoplastic urothelium with squamous differentiation. The MAC387 immunostaining consistently exhibited a diffuse and homogeneous pattern in the cytoplasm of macrophages.

In normal bladder tissue, macrophage infiltration was consistently low across all cases studied. In transitional cell carcinoma, macrophages were observed in both neoplastic urothelium and the tumor stroma, with a distribution that could be either diffuse or multifocal aggregates. The MAC387-positive macrophages were predominantly located within the tumor and its stromal areas. Seven cases (20.6%) showed low (score 1) TAM infiltration, twelve cases (35.3%) displayed moderate infiltration (score 2), and fifteen cases (44.1%) exhibited high infiltration (score 3).

**Figure 3:**
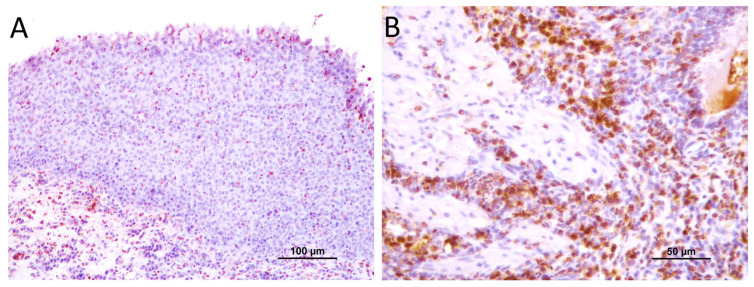
TAMs immunoexpression in canine TCC: (**A**) moderate TAMs (score 2) in low-grade transitional cell carcinoma; (**B**) high TAMs (score 3) in high-grade transitional cell carcinoma.

**Figure 4:**
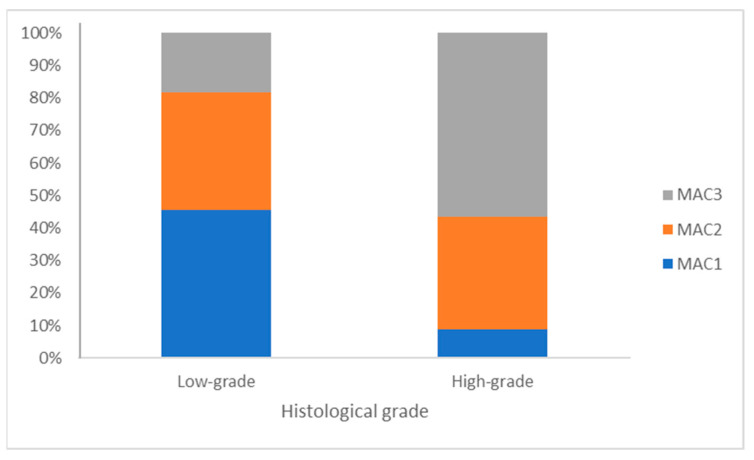
TAMs in high- and low-grade transitional cell carcinomas.

When considering the aggressiveness of the tumors, the immunohistochemical staining results indicated that the number of MAC387-positive macrophages was higher in high-grade tumor samples than in low-grade samples (*p* = 0.025). In low-grade malignant tumors, the majority presented with either low or moderate TAM counts (score 1 or 2), with only two low-grade tumors (18.2%) exhibiting high infiltration (score 3). In contrast, more than half of the high-grade malignant tumors displayed high infiltration by TAMs, with 2 out of 23 cases (8.7%) showing low infiltration and 8 cases (34.8%), thus demonstrating moderate infiltration (Figure 3 and Figure 4).

### 3.5. TAMs Are Not Statistically Associated with TATE

Most tumors exhibiting a low eosinophil count typically show a high presence of macrophages (11 out of 21) (Table 4). Conversely, tumors with many macrophages generally display a low count of eosinophils (11 out of 15). However, no statistically significant association was observed between TAMs and TATE. Tumors with a low macrophage presence can have either scarce (3/7) or abundant (3/7) TATE.

## 4. Discussion

TCC accounts for approximately 2% of all spontaneous cancer cases in dogs, making it the most prevalent urogenital cancer in this species. Around 20% of dogs show signs of metastasis at the time of diagnosis, with the presence of distant metastases associated with an unfavorable prognosis [5,40]. Given the aggressive nature of TCC and resistance to conventional therapies, identifying new therapeutic targets becomes essential.

Regarding clinical results, in our study, 47.1% of the animals were female, and 52.9% were male, contradicting the expectation of a higher incidence in females. The proportion of female and male dogs affected by TCC ranges from approximately 1.7 females for 1 male to almost 2 females for 1 male [10,41]. This may probably be due to their predisposition to urinary infections [3,4,5]. Inflammation, as a response to infections, initiates cellular changes and immunological responses, facilitating tissue repair and cell growth at the injury site. However, inflammation can become chronic if the underlying cause persists or regulatory mechanisms fail. The link between chronic inflammation and cancer is well recognized [40,42]. Chronic inflammation creates an environment with immune cells that can lead to cellular mutations and uncontrolled proliferative angiogenesis, creating an environment conducive to cancer development [43,44]. Chronic cystitis may increase the risk of transitional cancer in humans [45] and in dogs with polypoid cystitis [46]. Unfortunately, in our study, it was impossible to obtain accurate and consistent information about the clinical history of the animals, especially concerning any previous occurrence of cystitis.

Regarding dog breeds, our results showed that the mongrel (mixed breed) was the one with the highest number of cases (24 of 34), followed by the Poodle and Labrador Retriever. A breed-associated risk is noted in these tumors, particularly in Scottish Terriers, West Highland White Terriers, Shetland Sheepdogs, and Beagles, among others, compared to mixed breeds [1,2]. However, without knowledge of the most abundant breeds in the region, which may even be mixed-breed dogs, and with such a small number of cases, we cannot draw definitive conclusions. Interestingly, some cases occurred in Portuguese shepherd breeds such as Castro Laboreiro Dog and Estrela Mountain Dog. Given that the conclusions are limited due to the limited number of cases, it would be interesting, in the future, in extensive epidemiological studies, to discover the prevalence of tumors in these breeds and to search for a possible genetic or environmental association. The age of the animals with tumors was an average of 10.59 ± 2.85 years, according to values ranging from 9 to 11 years [2,4].

The main objective of this study was to investigate tumor-associated tissue eosinophilia (TATE) and tumor-associated macrophages (TAMs) in canine TCC, aiming to understand their association with tumor malignancy. In the absence of validated methods, the authors used the categories described in previous articles on canine tumors for evaluating these cells [37,39].

Eosinophils play important roles in innate and adaptive immunity, recognizing molecular patterns associated with pathogens and damage, leading to degranulation. In adaptive immunity, they help recruit other leukocytes, interact directly with them, and present antigens to T cells. In recent years, a role for eosinophils in the development and progression of tumors has been recognized, although results vary depending on studies and tumor type [47,48,49].

Using Congo Red staining, our results demonstrated that 21 cases (62%) had a low score of TATE. Regarding tumor malignancy, 87% of high-grade malignant tumors showed a lower score of eosinophilia. There are no studies on TCC in dogs to compare with our results. However, in canine squamous cell carcinoma, a higher number of eosinophils is associated with high histological differentiation of the tumor, potentially resulting in a less aggressive biological behavior, also consistent with our findings [37].

Considering human tumors, the involvement of eosinophils in cancer progression has also been suggested, highlighting the potential of these cells as prognostic markers [48,50,51]. The positive influence of eosinophils on prognosis has been evidenced not only in bladder cancer but also in other tumors, such as melanoma [30,52,53,54] and breast, ovarian [55], cervical [12], bladder [52], liver [56], gastric [57], esophageal [58], and colorectal tumors [59]. In a study on esophageal squamous cell carcinoma, eosinophils were associated with protection against cancer through the release of reactive oxygen species and the suppression of IL-17 [60]. Studies consistently indicate that tumor-associated eosinophilia is a favorable prognostic marker, showing an inverse association between eosinophil count and the metastatic potential of these neoplasms [30,61]. Although most of the studies described are in accordance with our results, TATE has also been associated with a poor prognosis in Hodgkin’s lymphoma and oral squamous cell carcinomas [47,62,63,64].

Thus, TATE may have a dual and contradictory role in the TME, as it can exhibit both anti-tumor and pro-tumor activity by stimulating or inhibiting the immune response [65,66]. Emerging data indicate that eosinophils infiltrate various solid tumors and manifest pleiotropic actions through at least two non-mutually exclusive mechanisms: direct interactions with tumor cells and complex cross-talk with other cells, such as the lymphocytes [67,68]. A Th1 or a combined Th1/Th2 immune response appears to be linked to tumor suppression through eosinophil activity, whereas an excessively activated type 2 response, which suppresses type 1 immunity, promotes tumor development. The role of eosinophils in determining the outcome of tumors is connected to the STAT signaling pathway. Phosphorylation of STAT1 impedes tumor growth, whereas STAT3 and STAT5 play roles in the initiation and development of tumors. Additionally, mediators released by eosinophils, such as IL-5, IL-33, CCL11 (eotaxin-1), granulocyte–macrophage colony-stimulating factor (GM-CSF), thymic stromal lymphopoietin (TSLP), interferon-gamma (IFN-γ), and tumor necrosis factor-alpha (TNF-α), play a significant role in the response of eosinophils to tumor cells [31,69,70].

Thus, our findings may raise awareness of the importance of immunotherapy, particularly regarding eosinophils. Immune checkpoint blockade and chimeric antigen receptor (CAR) T-cell treatments could be promising prospects [31]. In human patients with malignant melanoma treated with ICB, a high baseline eosinophil count is correlated with better overall survival (referring to the anti-PD1 antibody pembrolizumab) [71].

Our results might also indicate that considering eosinophil counts could be important in the routine histopathological evaluation of canine transitional cell carcinoma. Hence, additional investigations involving a larger sample size of tumors and prognostic assessments are essential to comprehend the true implications of our findings.

In cancer, TAMs play a crucial role at all stages, contributing to latent inflammation, a mutagenic environment, tumor progression, angiogenesis, cell migration, and invasion. Tumor progression results from the intricate interaction between tumor cells, mediated by cross-talk, where TAMs play pro- and anti-tumorigenic roles [72]. TAMs prepare the metastatic niche in metastases, facilitate extravasation, and increase tumor cell survival [15,73,74].

Our immunohistochemical analysis, using MAC 387 (a marker for reactive/infiltrating monocytes/macrophages [75]), revealed that 15 cases (44.1%) exhibited high TAM infiltration. Furthermore, the number of TAMs was higher in high-grade samples (*p* = 0.025) compared to low-grade samples. These findings align with the recent research on TAMs in canine transitional cell carcinoma. In 27 cases, an association with lung metastasis and prognosis was found [76]. Also, in human transitional cells, a high density of TAMs is associated with high-grade tumors and a worse prognosis [77,78,79].

TAMs can shift from the M1 state to the M2 state, the latter being linked to tumor growth, invasion, angiogenesis, metastasis, and immunosuppression. M1 macrophages, activated through the classical pathway by interferon-gamma (IFN-γ), are typically present in the tumor microenvironment (TME) during early stages of acute inflammation, promoting an anti-tumoral inflammatory response [17,66].

Upon activation through the alternative pathway, influenced by IL-4 and IL-13, TAMs differentiate into M2 subtypes (M2a, M2b, M2c, and M2d), depending on the activation mode. M2 macrophages, characterized by anti-inflammatory and pro-tumorigenic activity, secrete cytokines like IL-10 and TGF-β, contributing to a favorable tumor growth and progression environment. In summary, TAMs play a dynamic role in the TME, modulating their response from M1 to M2, significantly impacting the course of cancer and favoring conditions conducive to tumor development [80,81]. In solid tumors, including TCC, macrophages are attracted to the tumor site through chemotactic molecules such as CCL2 (MCP-1) and CSF-1 (M-CSF) [60,61]. Tumor cells release these chemotactic molecules to recruit macrophages, and the specific responses may vary depending on the tumor cells. For instance, bladder tumor cells release IL-6, IL-8, and other molecules, resulting in tumors with increased vascularization and infiltration. Understanding these mechanisms is crucial for therapeutic intervention [14,82].

Similar to TATE, our results might highlight the significance of immunotherapy, especially in relation to macrophages, such as blockers of CCL2–CCR2 or CCR5–CCL5 axes, macrophage checkpoint inhibitors, among others [23,24,26,27,28]. However, further studies are needed with a deeper characterization of the macrophage population associated with canine TCC of the bladder (M1, M2, and subpopulations) to better understand and identify effective therapeutic targets.

The present work also aimed to investigate whether there was a relationship between TAMs and TATE. Most tumors exhibiting a low eosinophil count typically show a high presence of macrophages, and tumors with many macrophages generally display a low count of eosinophils. These results suggest an inverse relation between these cells in canine TCC. Indeed, eosinophils can interact with immune cells, including macrophages, during cancer progression to induce anti-tumor responses [83]. This cross-talk could involve several mechanisms, including macrophage polarization [84]. However, our results lack a statistically significant relationship.

This study contributes to understanding the intricate relationship between macrophage and eosinophil infiltration and the aggressiveness of canine bladder carcinoma. Our findings suggest the connection between TATE and TAMs and tumor malignancy. Exploring the dynamics of these immune cells reveals promising markers for prognostic assessment in canine bladder tumors. These results could contribute to the growing knowledge about the role of the immune microenvironment in cancer progression. The observed associations with TAMs and TATE suggest potential avenues for targeted therapeutic interventions. By unraveling the complex interplay between immune cells and tumor characteristics, such as angiogenesis, metastasis, migration, and invasion, we are paving the way for more informed and effective treatment strategies for canine bladder TCC [85,86,87]. Recognizing TAMs and TATEs as promising prognostic indicators opens new possibilities for personalized and targeted approaches in treating this prevalent malignancy in canines.

## 5. Conclusions

Bladder cancer is a prevalent malignancy in both dogs and humans within the urinary tract. This study has demonstrated an association of TAMs with tumor malignancy and that an increase in TATE is associated with lower-grade tumors. TAMs and TATE could be promising prognostic tools and future therapies. Additionally, it is crucial to understand how these immune system cells influence cancer progression.

## Figures and Tables

**Table 1:** Histological grading system for canine transitional cell carcinoma of the bladder [33].

Histological Grade	Histological Features
Low-Grade	Organized proliferation of the urothelium, mild to moderate cellular atypia, rare cases of mitosis, and absence of invasion.
High-grade	Disorganized growth of the urothelium, loss of cell polarity, numerous mitotic events, presence of multiple nucleoli, and evidence of invasion.

**Table 2:** Evaluation criteria for TATE adapted from [37].

Infiltration	Number of TATE	Score
Low infiltration	0–10	1
Moderate infiltration	11–20	2
High infiltration	>21	3

**Table 3:** Evaluation criteria for TAMs adapted from [39].

Infiltration	Number of TAMs	Score
Low infiltration	<20	1
Moderate infiltration	20–100	2
High infiltration	>100	3

**Table 4:** Results related to the study on the association between TAMs and TATE.

Score	TATE 1	TATE 2	TATE 3	Total
TAM 1	3	1	3	7
TAM 2	7	2	3	12
TAM 3	11	2	2	15
Total	21	5	8	34

## Data Availability

The data can be obtained from the corresponding author upon reasonable request.
